# *Giardia duodenalis* and dysentery in Iron Age Jerusalem (7th–6th century BCE)

**DOI:** 10.1017/S0031182023000410

**Published:** 2023-07

**Authors:** Piers D. Mitchell, Tianyi Wang, Ya'akov Billig, Yuval Gadot, Peter Warnock, Dafna Langgut

**Affiliations:** 1 Department of Archaeology, University of Cambridge, Cambridge, UK; 2 Israel Antiquities Authority, Jerusalem, Israel; 3 Department of Archaeology and Ancient Near Eastern Cultures, Tel Aviv University, Tel Aviv, Israel; 4 Social Sciences Division, Muskegon Community College, Muskegon, MI, USA; 5 Laboratory of Archaeobotany and Ancient Environments, Institute of Archaeology & The Steinhardt Museum of Natural History, Tel Aviv University, Tel Aviv, Israel

**Keywords:** biblical archaeology, diarrhoea, dysentery, *Giardia duodenalis*, giardiasis, Kingdom of Judah, Near East, palaeoparasitology

## Abstract

The aim of this study was to determine if the protozoa that cause dysentery might have been present in Jerusalem, the capital of the Kingdom of Judah, during the Iron Age. Sediments from 2 latrines pertaining to this time period were obtained, 1 dating from the 7th century BCE and another from the 7th to early 6th century BCE. Microscopic investigations have previously shown that the users were infected by whipworm (*Trichuris trichiura*), roundworm (*Ascaris lumbricoides*), *Taenia* sp. tapeworm and pinworm (*Enterobius vermicularis*). However, the protozoa that cause dysentery are fragile and do not survive well in ancient samples in a form recognizable using light microscopy. Enzyme-linked immunosorbent assay kits designed to detect the antigens of *Entamoeba histolytica*, *Cryptosporidium* sp. and *Giardia duodenalis* were used. Results for *Entamoeba* and *Cryptosporidium* were negative, while *Giardia* was positive for both latrine sediments when the analysis was repeated three times. This provides our first microbiological evidence for infective diarrhoeal illnesses that would have affected the populations of the ancient near east. When we integrate descriptions from 2nd and 1st millennium BCE Mesopotamian medical texts, it seems likely that outbreaks of dysentery due to giardiasis may have caused ill health throughout early towns across the region.

## Graphical abstract

**Figure:**
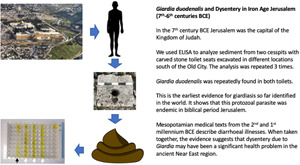

## Introduction

Infective diarrhoeal illness may be caused by pathogens such as viruses, bacteria and protozoan parasites. These are commonly spread by the contamination of water and food by human feces (Cairncross *et al*., 2010; Norman *et al*., 2010; Fink *et al*., 2011). While their health impact today is known to be significant, it is much more of a challenge to identify pathogens that cause diarrhoea and dysentery in past populations. Although the robust eggs of intestinal helminths have been shown to survive thousands of years in the remains of human feces from the near east (Harter-Lailheuge *et al*., 2005; Ledger *et al*., 2019; Mitchell, 2023), the fragile cysts of protozoa are easily deformed and damaged as feces decompose due to the action of soil microorganisms. This means they are extremely hard to detect using standard light microscopy. However, microscopy with immunofluorescent monoclonal antibodies (Faulkner *et al*., 1989; Le Bailly *et al*., 2008) and enzyme-linked immunosorbent assays (ELISAs) that use antibodies to detect antigens uniquely made by these protozoan organisms (Gonçalves *et al*., 2002, 2004; Le Bailly and Bouchet, 2006) have been found to be a successful way to detect these protozoa even when the cysts are damaged and deformed. This approach, therefore, allows us to search for early evidence for protozoan species that may have caused dysentery in ancient civilizations.

To date either ELISA or microscopy with immunofluorescence has successfully identified intestinal protozoan parasites in a range of early human populations. *Entamoeba histolytica* has been found in Neolithic Greece in samples from 5000 to 2000 BCE (Le Bailly and Bouchet, 2006) and *Cryptosporidium* sp. in 600–800 CE Mexico (Morrow and Reinhard, 2016). *Giardia duodenalis* has been identified in a 600–0 BCE coprolite from a cave in Tennessee, USA (Faulkner *et al*., 1989) and Roman period Turkey and Italy (2nd–5th century CE) (Williams *et al*., 2017; Ledger *et al*., 2021). Such evidence demonstrates how these species appear to have been successfully infecting humans in different regions of the world well into the past. However, much more research applying ELISAs to early societies is needed for us to fully understand from which regions of the world each organism originated, and when they spread to new areas due to migrations, trade and military invasions.

In medical texts from 2nd and 1st century millennium BCE Mesopotamia (ancient Iran and Iraq), the cuneiform word used to describe diarrhoea was sà si-sá. Diarrhoea is described in these texts as affecting infants and adults, and some texts describe incantations that they believed would help the sick person recover (Scurlock, 2014, 265; Steinert and Vacín, 2018). While these early written sources cannot allow us to differentiate the many causes of diarrhoea, they do encourage us to apply modern techniques to investigate which pathogens might have been involved.

The aim of this study was to investigate whether *G. duodenalis*, *E. histolytica* and *Cryptosporidium* sp. may have been present in the Near East region prior to the Roman period. The Near East is the region of the world where humans first created settlements, learned to farm and domesticate animals, and where the first large towns and cities developed (Bourke, 2018). As dysentery is more easily spread in environments with overcrowding, lack of organized sanitation and sewage systems, lack of understanding of how such diseases spread, and plenty of flies, we might expect the early cities of the Near East to have been well suited to disease outbreaks.

## Materials and methods

### Jerusalem in the 7th to early 6th century BCE

During this period of the Iron Age, Jerusalem was the capital of the Kingdom of Judah, a vassal kingdom under the yoke of the Assyrian empire until 630 BCE (Lipschits, 2021). While in the 9th century BCE Judah had been subservient to Aramean and then Neo-Assyrian neighbours, during the 8th and 7th centuries Jerusalem stood at its heart as a large, vibrant political and religious centre (Matthews, 2018, 128–164; Schipper, 2019, 45–70). Administrative apparatus expanded, as did ancient Hebrew literacy, Jerusalem expanded westwards, and the water supply for Jerusalem was improved (Gadot, 2022). In the 7th century BCE Jerusalem is estimated to have had between 8000 and 25 000 residents (Geva, 2014), with elite properties being built near the Temple Mount (Sapir-Hen *et al*., 2016; Shalev *et al*., 2020; Amir *et al*., 2022; Avisar *et al*., 2022). Towards the end of the 7th century BCE the Kingdom of Judah found itself between the competing powers of the Neo-Babylonians to the east and the Egyptians to the south, and paid tribute to each at different times. The Babylonian ruler Nebuchadnezzar II invaded the Kingdom of Judah and conquered Jerusalem in 598/597, and returned again and sacked the city in 587/586 when they refused to pay their agreed tribute (Matthews, 2018, 128–164; Schipper, 2019, 45–70; Vaknin *et al*., 2020; Lipschits, 2021).

### The 2 latrines

**Figure 1.**
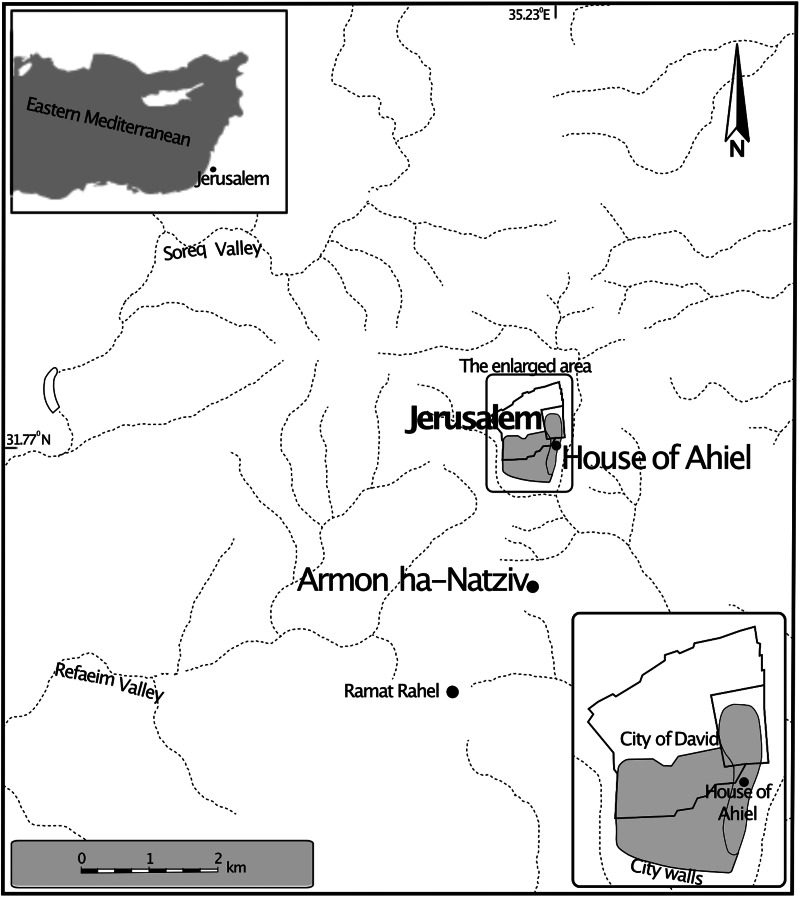
Map indicating location of House of Ahiel and Armon ha-Natziv, where the 2 Iron Age latrines were found at excavation. Image credit: Dafna Langgut.

**Figure 2.**
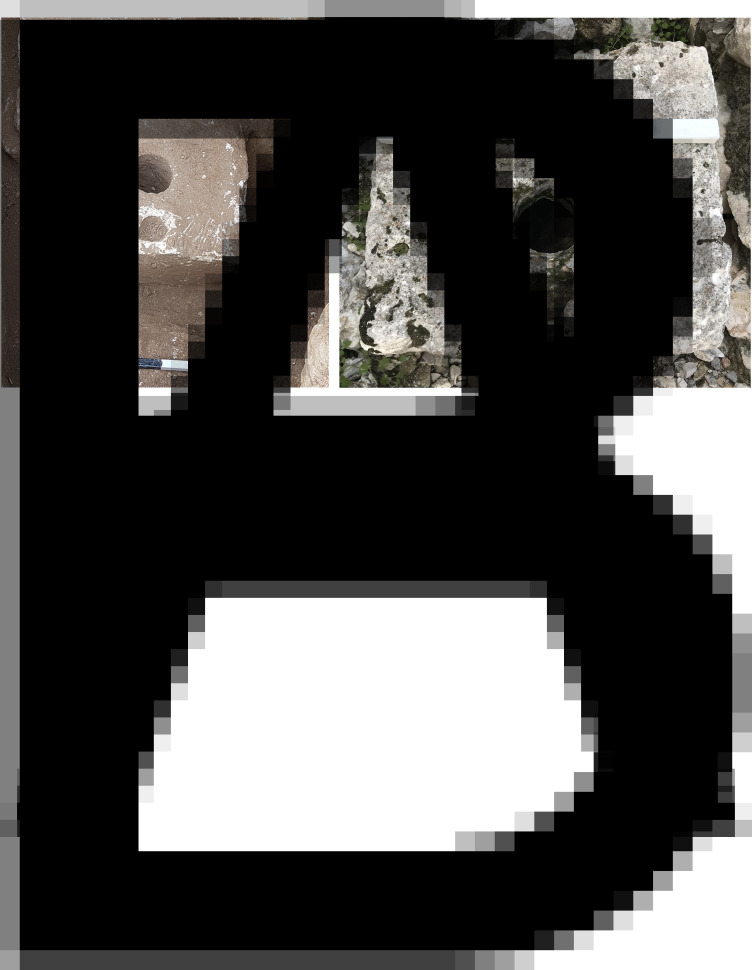
Stone toilet seats from Armon ha-Natziv (A, left) and House of Ahiel (B, right). Image credit: (A) Y. Billig, (B) F. Vukosavović.

In 2019–2020 a salvage excavation by the Israel Antiquities Authority at Armon ha-Natziv (south Jerusalem, Fig. 1) exposed an estate which included a collection of ornamented architectural elements made of soft limestone including medium-sized ‘Proto-Aeolian’ stone capitals, fragments of lavish window frames and balustrades made of stylish columns. The level of workmanship in these capitals is of the highest standard known to date in the southern Levant during the Iron Age (Billig, 2021; Billig *et al*., 2022). Based on ceramic typology, the site was dated to the mid-7th century BCE, probably the days of King Manasseh, who ruled over Judah for more than 50 years and was a client of the Assyrian empire (Gadot, 2022). According to Gadot the ornamental building that stood at the site should be understood as an Assyrian Bitanu. This suggestion is further supported by the evidence for an artificial garden (Langgut, 2022). It seems that the excavated area served as the garden of the estate and the actual building stood outside of the excavated lot. Within the estate garden a cubical stone object was found with a shallow curved surface for sitting, a large central hole for defecating and an adjacent hole likely for male urination (Fig. 2A). It was therefore interpreted as a stone toilet seat. It is thought that the toilet seat had fallen into the cesspit below after the floor support gave way, as stone slabs adjacent to the seat were steeply tilted downwards. Its dimensions are: 53 × 49 × 35 cm. Microscopy of sediment from this cesspit by Dafna Langgut has identified the eggs of whipworm, roundworm, *Taenia* sp. tapeworm and pinworm (Langgut, 2022).

The ‘House of Ahiel’ was a domestic building composed of 7 rooms, 4 of which form the main part of the building (Shiloh, 1984, 18 and Figs 20 and 25; Steiner, 2001). In room L789 a large locally carved block stone toilet seat was found (Fig. 2B) (Cahill *et al*., 1991), virtually identical in design to the Armon ha-Natziv toilet seat. The stone was positioned above a plastered cesspit and so seems to be positioned in its original place of use. The date of the construction of the House of Ahiel remains tentative at around the 8th century BCE, with some scholars suggesting an earlier date (Cahill, 2003). The destruction of the building is safely dated to 586 BCE, the Babylonian destruction of Jerusalem (Shiloh, 1984, 18). Microscopy of sediment from this cesspit by Karl Reinhard has identified the eggs of whipworm and *Taenia* sp. tapeworm (Cahill *et al*., 1991).

While the 2 toilets discussed in this paper are the only ones to have undergone parasite analysis, several other stone toilet seats from Late Iron Age II southern Levant have been found at excavation. Some are from the south eastern ridge of Jerusalem, known also as the City of David (Vincent, 1911, 29; Shiloh, 1984; Chapman, 1992; Steiner, 2001; De Groot and Bernick-Greenberg, 2012, 352; Vukosavović *et al*., 2021; Gibson, 2022). However, only the 2 installations that were reported by Shiloh were found *in situ*, above a cesspit (Cahill *et al*., 1991). The others were found out of context in various excavations (Vukosavović, in press, 2023). Two others were found at a fortress located at Zur Baher near Ramat Rahel (Eisenberg and De Groot, 2006), and 2 at the gate entrance of the main city of Lachish (Ganor and Kreimerman, 2019; Kleiman, 2020, Fig. 1).

### ELISA

A wide range of ELISA kits manufactured by different companies are available for the detection of the protozoa that cause dysentery (see e.g. Garcia and Shimizu, 1997; Van den Bossche *et al*., 2015). The ELISA kits we used were Entamoeba histolytica II™, Giardia II™ and Cryptosporidium II™ produced by TECHLAB^®^ (Blacksburg, Virginia, USA). Earlier research has indicated these tests typically have 96–100% sensitivity and specificity (Garcia and Shimizu, 1997; Boone *et al*., 1999).

One sample of sediment from the House of Ahiel cesspit was available for analysis, and 3 samples from different areas of the Armon ha-Natziv cesspit. A 1 g subsample of each was disaggregated using 0.5% trisodium phosphate solution, to form a suspension. This was passed through a stack of microsieves with mesh sizes 300, 160 and 20 *μ*m to remove large soil particles, and the material that passed through the 20 *μ*m sieve was used for ELISA analysis. This is because the cysts and oocysts of *Entamoeba*, *Giardia* and *Cryptosporidium* measure 5–19 *μ*m in diameter (Garcia, 2016). The microsieves were thoroughly cleaned in an ultrasonicator bath with detergent between each sample. The sieved suspension was then centrifuged to concentrate the volume required for the ELISA plates, in the process concentrating the component of the sediment that should contain the protozoa if present. Following the manufacturer's instructions, a positive and a negative control were included in each microassay plate. A column of 8 wells was used for each sample. An ELISA plate reader (BioTek Synergy HT, Santa Clara, California, USA) was set to 450 nm and used to generate the absorbance values. Positive and negative results were allocated following the manufacturer recommended absorbance values, with more than 0.150 absorbance value being positive. This analysis was repeated in its entirety (using different sediment subsamples) on 3 separate dates over the course of a 12 month period to ensure reproducibility of the results.

## Results

**Figure 3.**
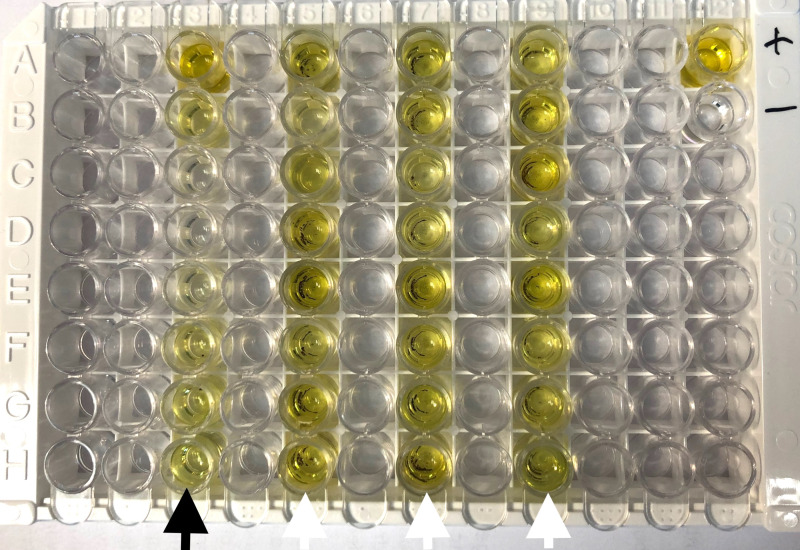
*Giardia* II ELISA microplate showing positive results in columns 3 (House of Ahiel, black arrow), and 5, 7, 9 (Armon ha-Natziv, white arrows). Image credit: Piers Mitchell.

Positive results from both latrine sediments were noted for *G. duodenalis* on the 3 dates the analysis was repeated. The House of Ahiel sample had between 2 out of 8 wells and 6 out of 8 wells positive on different analyses dates, while the 3 Armon ha-Natziv samples had all 8 wells positive each time the test was repeated. This might suggest that the Armon ha-Natziv cesspit sediment contained a higher concentration of *Giardia* antigen and cysts than did the House of Ahiel latrine, or that preservation of the antigen was better at the Armon ha-Natziv latrine. In contrast to the *Giardia* tests, the samples were negative for both *Cryptosporidium* sp. and *E. histolytica*. The *Giardia* ELISA plate values are given for each of the 3 analyses in Table 1, and image of the plate is shown in Fig. 3.

**Table 1.** ELISA absorbance values for *Giardia* II test kits

	First analysis	Second analysis	Third analysis
**House of Ahiel**	0.07	**0**.**15**	**1**.**87**
	0.08	**0**.**17**	**0**.**49**
	0.13	**0**.**16**	0.12
	**0**.**15**	**0**.**18**	0.11
	**0**.**42**	**0**.**17**	**0**.**22**
	0.13	**0**.**15**	**0**.**42**
	0.09	0.14	**0**.**64**
	0.13	0.11	**0**.**50**
**Armon ha-Natziv**	**0**.**41**	**0**.**16**	**0**.**84**
Sample 5 p/p	**0**.**47**	**0**.**26**	**0**.**28**
	**0**.**43**	**0**.**22**	**0**.**70**
	**0**.**43**	**0**.**18**	**0**.**86**
	**0**.**35**	**0**.**18**	**1**.**14**
	**0**.**53**	**0**.**20**	**0**.**54**
	**0**.**51**	**0**.**18**	**0**.**85**
	**0**.**54**	**0**.**17**	**0**.**84**
Sample 6 p/p	**0**.**44**	**0**.**45**	**0**.**86**
	**0**.**44**	**0**.**44**	**0**.**73**
	**0**.**53**	**0**.**45**	**0**.**40**
	**0**.**55**	**0**.**36**	**0**.**51**
	**0**.**61**	**0**.**34**	**1**.**01**
	**0**.**51**	**0**.**24**	**0**.**75**
	**0**.**52**	**0**.**24**	**0**.**83**
	**0**.**52**	**0**.**30**	**0**.**82**
Sample 12 p/p	**0**.**43**	**0**.**43**	**0**.**63**
	**0**.**44**	**0**.**50**	**0**.**84**
	**0**.**55**	**0**.**54**	**1**.**10**
	**0**.**53**	**0**.**37**	**0**.**56**
	**0**.**49**	**0**.**54**	**1**.**09**
	**0**.**40**	**0**.**43**	**0**.**58**
	**0**.**52**	**0**.**49**	**0**.**63**
	**0**.**52**	**0**.**40**	**0**.**90**

Positive readings are those measuring 0.150 and above, and are highlighted in bold.

## Discussion

The results presented here give what is currently the earliest known evidence for *G. duodenalis* (syn. *G. lamblia*, *G. intestinalis*, *G. enterica*) so far identified in a past population anywhere in the world. It has previously been identified in Roman period Turkey and also in Israel during the medieval and Ottoman periods (Mitchell *et al*., 2008; Yeh *et al*., 2015; Williams *et al*., 2017; Eskew *et al*., 2019). In light of this, these results from Iron Age Jerusalem likely indicate the long-term presence of this parasite in the populations of the Near East.

### Reliability of the result

In view of the significance of this result, we should carefully explore the reliability of this analysis. The latrines were clearly identified as such by the presence of toilet seats. The samples were taken from the cesspit beneath each seat by the archaeologists excavating latrine, with each latrine excavated by different archaeologists some decades apart. The cesspit sediment from each latrine was found to contain intestinal helminth eggs on microscopy (Cahill *et al*., 1991; Langgut, 2022). They were no longer used after the destruction of Jerusalem by the Babylonians in 586 BCE. Therefore, it is highly unlikely that the sampled sediment was contaminated by the environmental conditions or by those excavating the site.

The Techlab *Giardia* II ELISA kits use monoclonal and polyclonal antibodies to detect the cyst wall protein 1 (CWP1), which is a stable protein produced and released by encysting *Giardia* trophozoites (Boone *et al*., 1999). No test is completely accurate all the time, so an understanding of the limitations of the tests used here is helpful. In a study using fresh stool microscopy, polymerase chain reaction and the Techlab *Giardia* II kit, sensitivity was 97% and specificity was 100% for the ELISA kit (Silva *et al*., 2016). Another study based in 3 separate institutions that used fresh stool microscopy and ELISA found the Techlab *Giardia* II kit to have 91–100% sensitivity, and 97.8–100% specificity, depending on the institution (Boone *et al*., 1999). In other words, the test may sometimes miss a true infection (perhaps if cyst concentrations are low), but a positive result is highly likely to be genuine. We do accept that it is theoretically possible that detection of coproantigens such as CWP1 could be confounded by cross-reactions with site-specific environmental antigens. However, our ability to cross-check with alternative molecular methods is outside the scope of our current investigation. The fact that we found samples from both Iron Age latrines to be positive on repeating the entire analysis process three times on separate dates is reassuring. On our last test run we left empty columns (plate columns 2, 4, 6, 8, 10) between the Iron Age samples (plate columns 3, 5, 7, 9) to ensure there was no contamination from 1 column to the next during the microassay plate washing required for various steps of the analysis, and the columns between the Iron Age samples gave a clear negative result (see Fig. 3). This again is reassuring.

### Implications for our understanding of ancient populations of the Near East

*Giardia* is a flagellated protozoan parasite that lives in the small intestine as a pear-shaped trophozoite measuring 9–20 *μ*m in size, and as an oval infectious cyst that typically measures 8–12 *μ*m. Recent assessment of the gene sequences of *Giardia* species complex has noted a number of distinct host-specific assemblages (Wielinga *et al*., 2023), but detection using ELISA is not able to differentiate each assemblage. *Giardia* is spread by the contamination of water or food with the feces of an infected person or non-human mammal. Trophozoites attach themselves to the lining of the intestine, which results in inflammation and damage to the epithelium and microvilli. Symptomatic infection by *Giardia* is termed giardiasis. Common symptoms include diarrhoea, abdominal cramps, malabsorption and weight loss. However, not all infections cause symptoms (Ryan *et al*., 2019; Adam, 2021). Many individuals fully recover after an acute infection, but up to a third can experience chronic diseases such as post-infective irritable bowel, ocular pathology, arthritis, allergies and muscular complications. Most of those who die from *Giardia* are children, and chronic infection in this group can lead to stunted growth, impaired cognitive function and failure to thrive (Halliez and Buret, 2013).

The fact that the sediment from both Iron Age cesspits was positive for *Giardia* would suggest that this parasite was endemic in the region of Jerusalem and the Kingdom of Judah during the 7th to early 6th century BCE. Since there was trade and military expeditions taking place across the Near East throughout this time period, we would expect such gastrointestinal infections to be spread easily by travellers. The many large and crowded towns and cities existing across the Near East by this time would have been fertile areas for the spread of such infections. While they did have toilets with cesspits across the region by the Iron Age, they were relatively rare and often only made for the elite. Towns were not planned and built with a sewerage network, flushing toilets had yet to be invented and the population had no understanding of existence of micro-organisms and how they can be spread (McMahon, 2015). Furthermore, the house fly (*Musca domestica*) is widespread in the Near East, and is well known for its ability to spread enteric pathogens that cause diarrhoea (Bidawid *et al*., 1978; Cohen *et al*., 1991). Therefore, it is probable that such flies contributed to the spread of diarrhoeal illness in the ancient Near East as well. It seems that at least some of those descriptions of diarrhoea in 2nd and 1st millennium BCE Mesopotamian medical texts (Scurlock, 2014, 265; Steinert and Vacín, 2018) may well have included individuals suffering with giardiasis.

### The evolutionary origins of *G. duodenalis*

With these results expanding our knowledge of early *Giardia* infection in humans, we can consider how this impacts our understanding of the role of giardiasis in human evolution (Mitchell, 2013). *Giardia* has now been found in early human feces from the Near East (7th century BCE), and early samples from North America (600–0 BCE) (Faulkner *et al*., 1989). While humans evolved in East Africa, so far *Giardia* has not been found in early human populations there, such as ancient Egypt (Anastasiou and Mitchell, 2015). We should ask whether this might indicate that *Giardia* was not present in Africa during human evolution, or is it just that archaeological evidence may have failed to survive in samples from that continent, or that samples have not been tested with techniques likely to be successful in its detection (which is certainly an issue). As *Giardia* is a zoonotic parasite that can affect humans and other mammals (Ledger and Mitchell, 2022), if it were to be known to be endemic in wild non-human primates in Africa, this would be more suggestive of its long-term involvement in the human evolutionary tree. In fact, *Giardia* has been identified in modern wild populations of Red Colobus monkeys, gorillas and less frequently chimpanzees (Ashford *et al*., 2000; Gillespie and Chapman, 2008; Brynildsrud *et al*., 2018). So unless these reflect recent infection of wild primate populations by humans visiting their forests, this third piece of evidence would support the suggestion that *Giardia* may have been an heirloom parasite that infected humans and other mammals throughout our evolution, and was spread around the planet with migrations to new regions.

## Conclusion

This study investigates some of the causes for infectious diarrhoea in the early populations of the Near East. Using sediment from 2 cesspits, we focused on the population of Iron Age Jerusalem, which was the capital of the Kingdom of Judah. Since both latrine sediments gave positive results for *G. duodenalis*, this would indicate that giardiasis was endemic in the region during the 7th to early 6th century BCE. Having explored aspects of life in the towns and cities of the ancient Near East that might predispose the population to infection by infective diarrhoeal illness, we conclude that the limited sanitation technologies available at the time, the shortage of fresh water for much of the year, the population density of these towns and widespread house flies all had the potential to contribute to infection. When we consider this evidence in the light of the textual descriptions of diarrhoea in medical texts from the 2nd and 1st millennium BCE Mesopotamia, we gain a fascinating insight into health and disease of the early populations of biblical period Jerusalem and indeed the entire ancient Near East.

## Data Availability

All the raw data relevant to this paper is given in table 1.
